# Analysis of motives and patient satisfaction in oncological second opinions provided by a certified university breast and gynecological cancer center

**DOI:** 10.1007/s00404-020-05525-2

**Published:** 2020-04-09

**Authors:** Christian R. Loehberg, Julia Meyer, Lothar Häberle, Carolin C. Hack, Sebastian Jud, Alexander Hein, Marius Wunderle, Julius Emons, Paul Gass, Peter A. Fasching, Sainab Egloffstein, Jessica Krebs, Yesim Erim, Matthias W. Beckmann, Michael P. Lux, Sonja Wasner

**Affiliations:** 1 Department of Gynecology, Erlangen University Hospital, University Breast Center and University Gynecological Cancer Center for Franconia, Universitaetsstr. 21-23, 91054 Erlangen, Germany; 2 Office of the CCC Erlangen-EMN (Comprehensive Cancer Center of the European Metropolitan Region of Nuremberg-Erlangen), Erlangen, Germany; 3 Psychosomatic and Psychotherapeutic Department, Erlangen University Hospital, Erlangen, Germany; 4 Breast and Gynecological Cancer Center, Women´s Hospital St. Louise, Paderborn, Women´s Hospital St. Josefs-Krankenhaus, Salzkotten, Frauen- und Kinderklinik St. Louise, St. Vincenz Hospital, Paderborn, Germany

**Keywords:** Second opinion, Breast cancer, Gynecological malignancy, Oncology center, Doctor–patient communication, Patient satisfaction

## Abstract

**Purpose:**

Second opinions in oncology are becoming increasingly important in an era of more complex treatments and a growing demand for information by patients. Therefore, we analyzed their effects and influencing factors like patients’ motives, subjective extent of information and satisfaction with communications.

**Methods:**

This prospective study evaluated second opinions for patients with breast cancer or gynecological malignancy. The patients received a questionnaire before and two months after, which inquired expectations, reasons, and satisfaction with the second opinion and the attending physicians.

**Results:**

A total of 164 patients were included and the majority had breast cancer (75.0%). Receiving the second opinion made 89.7% feel better informed, their need for information decreased (from 75.3% to 39.2%, *P* < 0.0001), and satisfaction with doctor–patient communications increased (from 61.9 to 91.8%, *P* = 0.0002). There were various reasons for requesting a second opinion, e.g., the extremely stressful situation of a cancer diagnosis, hope for change in the treatment recommendation or dissatisfaction with the initial physicians.

**Conclusions:**

Second opinions can lead to significantly greater patient satisfaction, meeting the need for information and leading to better management of patients in the extremely stressful situation of a cancer diagnosis. Doctor–patient communications play a key role.

**Electronic supplementary material:**

The online version of this article (10.1007/s00404-020-05525-2) contains supplementary material, which is available to authorized users.

## Introduction

An initial diagnosis of cancer leads many patients to carry out careful research on the diagnosis and the best treatment approach for it. In a large number of cases, however, patients only turn to a certified organ cancer center or oncology center when there are difficulties or setbacks—for example, immediately after an intervention, or when a relapse or metastasis occurs. However, adequate interdisciplinary consultation and care within the structures provided by certified centers would already be advisable at the time of the initial diagnosis [1].

The establishment of oncological centers and certification procedures conducted by the German Cancer Society (*Deutsche Krebsgesellschaft e. V.,* DKG) is promoting optimization of the quality of care for patients diagnosed with cancer and is encouraging interdisciplinary collaboration [2]. With its certified Breast and Gynecological Cancer Center [3, 4], the Department of Gynecology at Erlangen University Hospital forms part of the university’s Oncological Center and is also part of an interdisciplinary Oncological Center of Excellence, the Comprehensive Cancer Center Erlangen–European Metropolitan Region of Nuremberg (CCC Erlangen-EMN).

Within this framework, various organ-specific tumor conferences, including an interdisciplinary tumor conference at the certified Breast and Gynecological Cancer Center, have made it possible to provide care for a large number of individual oncological cases within short periods of time, with involvement of multidisciplinary specialist expertise for individual treatment recommendations [5–9]. Ideally, the results of the interdisciplinary tumor conference can be based on complete and current staging findings, as well as the current status of the patient’s clinical and physical findings and her psychosocial situation, thus leading to a sound and evidence-based assessment of the overall situation, taking into account all of the individually decisive factors [1, 10].

In addition to treatment planning for the Department’s own patients, the Department of Gynecology at Erlangen University Hospital is also increasingly providing advice to external institutions and patients, offering oncological second opinions as a regional and nationwide reference center. This has been particularly the case since 2014, when Erlangen University Hospital began taking part in a pilot project organized by the statutory health-insurance scheme in Germany (*Allgemeine Ortskrankenkassen*, AOK) in accordance with the country’s Social Security Code (*Sozialgesetzbuch,* SGB V paragraphs 63 ff.), providing for medical second opinions in an interdisciplinary tumor board for patients with oncological diseases.

A second opinion is defined as obtaining a second treatment proposal after a suggested treatment has already been presented by another physician [11]. The second opinion can confirm the first opinion or offer alternatives and discuss the advantages and disadvantages of each treatment option in detail. Patients’ reasons for obtaining an oncological second opinion may vary widely, and depending on the published report and the country concerned are very heterogeneous. A major motivation is often the hope that the diagnosis or proposed treatment options in the first opinion will differ from the second opinion, particularly to avoid over- or undertreatment. Another important reason is dissatisfaction with communications between patients and their initial physicians [12–16].

The pilot project mentioned above also included prospective scientific monitoring and analysis of various aspects of second opinions in oncological diseases. In a subsample of patients, our study group surveyed levels of mental resilience, dysfunctional fear of progression and distress levels as possible motivators for the search of a second opinion [17]. We found high levels of fear of progression and psychosocial distress, but concerning resilience, the patients were comparable to the norm. The aim of the present survey was to analyze second opinions in oncology in relation to their frequency, influencing factors, and characteristics in patients with breast cancer or gynecological malignancies. Particular attention was given to patients’ motives, the subjective extent of their information, and their satisfaction with communications with the attending physicians.

## Patients and methods

### Study design

In this prospective study, data were collected from patients who received an oncological second opinion with presentation at the interdisciplinary tumor conference in the Department of Gynecology at Erlangen University Hospital in the period from June 2014 to September 2016. Patient questionnaires were used to evaluate aspects of the oncological second opinion, proposed treatments, doctor–patient communications, patient satisfaction, and psycho-oncological issues.

The study was approved on September 10, 2013 by the Ethics Committee of the Medical Faculty of Friedrich Alexander University of Erlangen–Nuremberg (ethics vote no. 175_13B).

### Recruitment and inclusion criteria

The patients generally had two ways of obtaining an oncological second opinion. Patients with breast cancer or a gynecological malignancy could either present directly for a second opinion during the special consultation hours in the Department of Gynecology at Erlangen University Hospital, or they could request a second opinion by phone through the “CCC Erlangen–EMN Cancer Information” service at Erlangen University Hospital, which subsequently arranged for presentation of the case at the relevant interdisciplinary tumor conference. If registration for a second opinion was made directly through the special consultation hours in the Department, the patients received a consent form and patient questionnaire on site (time T0), and completed them. If patients opted for arrangement by the CCC Erlangen-EMN Cancer Information service, the consent form and patient questionnaire were sent to them by post. In both cases, the patients received the questionnaires before presentation at the interdisciplinary tumor conference. Subsequently, a follow-up questionnaire was sent to the patients after an interval of at least two months after receipt of the second opinion (time T1). If patients did not return the follow-up questionnaire, they were contacted either by phone or by post.

During the period investigated, 488 patients in the Department of Gynecology at Erlangen University Hospital received an oncological second opinion with presentation at the interdisciplinary tumor conference. A total of 167 patients agreed to participate in the study, three of whom had to be excluded as the case did not represent an oncological second opinion according to the following inclusion criteria:

- Active request for an oncological second opinion.
- Adult patients of any age and sex.
- Fully completed and signed declaration of consent.
- Presence or diagnostic clarification of suspected breast cancer or gynecological malignancy.
- Presentation at the interdisciplinary tumor conference in the certified breast cancer or gynecological cancer center in the Department of Gynecology at Erlangen University Hospital.

A total of 97 of the 164 patients (59.1%) responded to the follow-up questionnaire (time T1) two months after receiving the second opinion.

The patients’ age was defined as age at the time of first contact, or at the time when the patient questionnaire was received by post. The date of the initial diagnosis was determined on the basis of the date of the histological findings. If this was known from outside sources, the date was adopted. Staging of tumor entities was performed in accordance with the seventh edition of the TNM classification [18].

### Patient questionnaires

The patient questionnaires used were developed specifically for the study (see patient questionnaires (in German language) as supplement). The first patient questionnaire (time T0) collected general sociodemographic data for the patients and information about the tumor and previous treatments (including aspects of complementary and alternative medicine). It also inquired into motives, expectations, etc. regarding the second opinion, satisfaction with the physicians initially treating the case (especially with regard to physician–patient communications), and psychological states.

The follow-up questionnaire (time T1) inquired into the same aspects after the second opinion had been given, particularly in relation to possible changes, patient satisfaction with the second opinion or second-opinion physicians, doctor–patient communications, and information needs. Finally, the patients were asked to sum up their conclusion.

Supplementary information about tumor entity, menopausal status, prior treatments, and date of birth was obtained from the clinical information system (Soarian Clinicals^®^) at Erlangen University Hospital during analysis of the written or digital patient file.

### Statistical analysis

The descriptive data were presented using absolute and relative frequencies and median with minimum and maximum. Satisfaction with physicians was analyzed using logistic regression. The predictors used for the logistic regression were: “Did the first attending physician take enough time?”; “Is there any hope for a change in the diagnosis?”; “Is there any hope for a change in the treatment?”; and “Is further treatment planned in the Department of Gynecology at Erlangen University Hospital?” (each yes/no). Odds ratios with 95% confidence intervals (CI) and *P* values were calculated. Patients were repeatedly divided into two groups in relation to their motive for requesting a second opinion. The group assignment was based on binary variables representing the person initiating the request for a second opinion (patient herself, her partner, children, friends, family physician, gynecologist, physician). For each of these groupings, the patient’s age was compared using the *t* test and mean values and 95% confidence intervals were determined. Changes in patient’s information requirements and satisfaction with doctor–patient communications between time T0 and time T1 were analyzed using the McNemar test. As a sensitivity analysis, we divided the patients by disease situation (curative vs. palliative) as well as by cancer type (breast cancer vs. gynecological malignancies) and compared reasons for deciding to seek a second opinion via Pearson's chi-squared test. All of the tests were two tailed, with a significance level of *α* = 0.05. Correction of the *P* values due to multiple testing was not performed. The R statistical package (version 3.4.0, 2017, R Core Team, Vienna, Austria) and IBM SPSS^®^ Statistics (version 24) were used for the analyses.

## Results

### Description of the study group

The study included 164 patients with breast cancer or gynecological malignancies who received an oncological second opinion, agreed to participate in the study, and completed at least the first patient questionnaire (time T0) in the period from June 2014 to September 2016. Of these patients, 97 returned the follow-up questionnaire (time T1), representing a response rate of 59.1%.

The median age in the study group was 50.9 years (minimum 19.4 years, maximum 83.2 years) and all of the patients recruited were female. Most of the patients had children (75.6%, *n* = 124), were married (64.0%, *n*  = 105), were of German origin (92.7%, *n* = 152), and had an intermediate or high educational level (72.6%, *n* = 119)—i.e., at least an intermediate school-leaving certificate.

The majority of the patients (75.0%, *n* = 123) had breast cancer (or papilloma, at 1.2%, *n* = 2). The second most common carcinoma was endometrial carcinoma, at 9.8% (*n* = 16), followed by other gynecological malignancies (cervical carcinoma 7.3%, *n* = 12; ovarian carcinoma 5.5%, *n* = 9; vulvar carcinoma 1.2%, *n* = 2; tubal carcinoma 0.6%, *n* = 1). One patient had two gynecological malignancies (endometrial and cervical cancer).

Analysis of the disease situation showed that the majority of the patients were requesting a second opinion in the curative disease situation [adjuvant 64.6% of the patients (*n* = 106) or neoadjuvant 4.9% of the patients (*n* = 8)]; while, 30.5% (*n* = 50) were presenting for an oncological second opinion when the disease was already locally advanced or metastases were present (palliative).

### Reasons for seeking a second opinion

The patient questionnaire at time T0 was used to assess the reasons why the patients surveyed (*n* = 164) had decided to seek an oncological second opinion. Specific questions were asked (multiple answers and free-text answers were possible). Most frequently, the patients stated that they “wanted to leave no stone unturned” in their cancer treatment (85.4%, *n* = 140) and that the “opinion of several physicians would lead to the best therapy” (84.1%, *n* = 138).

In all, 62.2% of the patients (*n* = 102) felt “extremely stressed” by the “exceptional situation,” but only 23.8% (*n* = 39) were “not satisfied with the first doctor.” Additional reasons for deciding to seek a second opinion are listed in Table 1.

**Table 1:** Patients’ reasons for deciding to obtain a second opinion (multiple answers possible, *n* = 164)

	Frequency (*n*)	%
Poor state of health	45	27.4
Extremely stressful situation	102	62.2
Hope for a change in the diagnosis	49	29.9
Hope for a change in therapy	92	56.1
Hope for confirmation of the diagnosis	85	51.8
Hope for confirmation of therapy	88	53.7
Fear of making the wrong decision	126	76.8
Not leaving any stones unturned	140	85.4
Optimal treatment due to opinions from several doctors	138	84.1
Dissatisfaction with the initial physician	39	23.8

In a sensitivity analysis divided by disease situation, patients in the palliative situation stated significantly more often “extremely stressful situation” (84.4% vs. 61.5%, Pearson's Chi-squared test *P* = 0.01), “poor state of health” (63.8% vs. 14.6%, Pearson's Chi-squared test *P* < 0.001), and “hope for confirmation of therapy” (71.1% vs. 26.0%, Pearson's Chi-squared test *P* < 0.001) as reasons for deciding to seek a second opinion compared to patients in the curative situation.

In view to the examined aspects of seeking a second opinion, there were no significant differences among patients with breast cancer or gynecological malignancies except for an increased “hope for a change in therapy” (82.9% vs. 57.8%, Pearson's Chi-squared test *P* = 0.01) in patients with a gynecological malignancy.

### Initiator for a second opinion

Another point assessed in the patient questionnaire at time T0 was who had suggested or initiated the request for a second opinion. It was the patient herself in 67.7% of cases (*n* = 111), while her partner was the initiator for more than a quarter of the patients (Table 2).

**Table 2:** Initiator for obtaining a second opinion (multiple answers possible, *n* = 164)

	Frequency (*n*)	%
Patient herself	111	67.7
Partner	43	26.2
Children	31	18.9
Friends	27	16.5
Family physician	20	12.2
Gynecologist	29	17.7
Other doctors	21	12.8
Other	6	3.7

The analysis also examined whether there were any age differences in relation to motives for requesting an oncological second opinion. Patients who independently decided to seek a second opinion were significantly younger (50.7 years; 95% CI 48.3–53.1 years) than patients who were motivated to request a second opinion in other ways (55.4 years; 95% CI 51.9–58.9 years; *t* test *P* = 0.04). In contrast, patients for whom it was their children who suggested they should seek a second opinion were significantly older (49.8 years, 95% CI 47.8–51.9 years vs. 62.1 years, 95% CI 57.8–66.3 years; *P* < 0.001), as were patients who were encouraged by their family physician (51.3 years, 95% CI 49.2–53.4 years vs. 58.6 years, 95% CI 53.0–64.2 years; *P* = 0.03). There were no significant age differences among patients for whom it was their friends, partners, gynecologists, or other physicians who initiated a desire for a second opinion. The study also examined whether disease situation, cancer type or educational level influenced an increased need for information in our study group, but no significant correlations were found.

### Influences on satisfaction with the initial physician

In addition, the questionnaire at time T0 inquired about patients’ satisfaction with the physician initially treating them. It was found that almost 70% (*n* = 114) were quite satisfied with the doctors who provided initial treatment, but 73.2% (*n* = 120) of the patients still had a need for further information. Table 3 summarizes other points relating to satisfaction with initial physicians.

**Table 3:** Patients’ satisfaction with the physician initially treating them (multiple answers possible, *n* = 164)

	Answer “yes” (*n*)	%
Did the first doctor take enough time?	37	22.6
Was the doctor who first treated you sensitive?	105	64.0
Was the first doctor annoyed that you asked for a second opinion?	23	14.0
Were you satisfied with communications with him or her?	106	64.6
Do you need further information?	120	73.2
Are you generally satisfied with the doctor who treated you the first time?	114	69.5

Whether the first physician “took enough time” had a significant influence on satisfaction (OR 0.05; 95% CI 0.02–0.16; *P* < 0.001). Hoping for a change in the diagnosis did not have any significant influence on patients’ satisfaction with the first physician, nor did hoping for a change in treatment or whether or not further treatment in the Department of Gynecology at Erlangen University Hospital was planned.

Using the follow-up questionnaire (time T1; *n* = 97), the patients were also asked about their satisfaction with the first physician two months after they had received the second opinion, to assess whether their relationship with the first physician had changed as a result of the second opinion. More than half of the patients (*n* = 53) stated that obtaining a second opinion had no influence on their relationship with the first doctor (Table 4). However, 27.8% of the patients had less confidence or no confidence in the doctors who had treated them initially.

**Table 4:** Influence of obtaining a second opinion on the doctor–patient relationship (*n* = 97)

	Frequency (*n*)	%
No influence on the relationship	53	54.6
More confidence in the doctor	3	3.1
Less trust in the doctor	16	16.5
No more trust in the doctor	11	11.3
Other	9	9.3
Not specified	5	5.2

### Subjective level of information and satisfaction with doctor–patient communications after a second opinion

Further analysis of the responses (*n* = 97) in the follow-up questionnaire (time T1) showed that 89.7% of the patients (*n* = 87) felt better informed after the second opinion and that their “need for further information” decreased significantly after the second opinion. Whereas the majority of patients (75.3%, *n* = 73) stated before the second opinion that they had a “need for further information,” only 39.2% of them (*n* = 38) still needed further information after the second opinion (McNemar test, *P* < 0.0001).

**Fig. 1:**
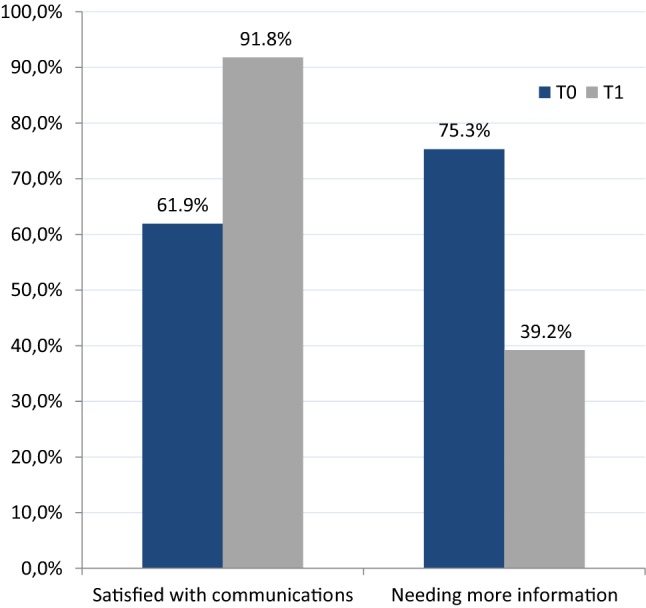
Changes in patient satisfaction with doctor–patient communications (*P* = 0.0002) and the need for information (*P* < 0.0001) before obtaining the second opinion (time T0) and 2 months afterwards (time T1, *n* = 97)

In contrast, satisfaction with doctor–patient communications increased from just under 62% (*n* = 60) to 92% (*n* = 89; *P* = 0.0002) as a result of the second opinion, and the majority of patients were very satisfied with communications between them and the consulting physicians for the oncological second opinion (Fig. 1).

## Discussion

Hardly any current data, and particularly results from prospective studies, are available concerning oncological second opinions and their effects on treatment success, patient satisfaction, and the doctor–patient relationship [19, 20]. This prospective study shows that the majority of patients (89.7%) felt better informed as a result of the second opinion—an essential basis for adequate treatment decision-making after weighing up the individual advantages and disadvantages.

Among the major reasons patients gave for obtaining a second opinion were the extremely stressful situation of a cancer diagnosis (62.2%), especially in the palliative situation, or that they were hoping for a change in the treatment recommendation due to the second opinion (56.1%), which especially applied to patients with a gynecological malignancy. Furthermore, about a quarter of patients (23.8%) were dissatisfied with the physicians who first treated them. Age was also an important factor behind obtaining a second opinion. Patients with children who prompted them to seek a second opinion were significantly older than patients motivated to obtain a second opinion for other reasons.

The present study group and the data presented are consistent with other research studies on oncological second opinions, in which it was shown that the typical second-opinion patient is female, about 50 years old, and has breast cancer [13–16]. The reasons reported for obtaining a second opinion in these studies were in some cases different, but the hope that first and second opinions might differ was often a major motivation. In addition, some patients requested a second opinion out of dissatisfaction with communications between them and the initial physicians [12]. Many patients wanted a second specialist opinion in view of the exceptional situation they were in due to their cancer, and some of the patients interviewed obtained a second opinion to confirm that the first physician’s diagnosis and/or proposed treatment options were correct, so that they were reassured about the diagnosis and proposed treatment options [13–16].

Nearly 92% of the patients in the present study were satisfied with the way in which the oncological second opinion was communicated, and 89.7% of the patients felt better informed afterwards. This aspect plays an important role in oncological treatment approaches. Good doctor–patient communications significantly improve not only patient satisfaction, but also patient compliance during therapy. This was confirmed by a meta-analysis including 106 studies and 21 experimental interventions [21]. Patients who did not have satisfactory communication with the physicians treating them were at significantly higher risk of not adhering to the suggested treatments or not being able to complete their therapy [21]

When a therapy is being chosen, patients’ preferences should also be taken into account, so that the final decision is made jointly by the doctor and patient [12]. However, since this type of doctor–patient communication does not yet actually take place to a sufficient extent in practice, an approach involving obtaining an oncological second opinion before important therapy decisions are made can be recommended [1].

Even at the end of the 1990s in Germany, section 11 of Social Security Code (SGB) V did not list obtaining a second opinion as a benefit that was reimbursable under statutory health insurance. As a result, patients often had to pay for a second opinion out of their own pockets. On July 23, 2015, a Law on Enhancing Care Provision under Statutory Health Insurance (*GKV-Versorgungsstärkungsgesetz*) came into force in Germany [22]. This was intended to subsequently ensure medical care at the highest level that would be accessible to everyone. In addition to regulations on better care for underserved rural areas, on fairer appointment allocation, and on the promotion of health-care research, people with statutory insurance are entitled to obtain a second medical opinion before certain interventions, so that only medically necessary interventions are carried out and over- or undertreatment is avoided [19, 20, 22]. Although the implementation of the Law on Enhancing Care Provision under Statutory Health Insurance, at least in the first phase, was initially related to second opinions particularly in non-oncological surgery, the German Cancer Society (DKG) launched a pilot project in April 2019 on medical second opinions for patients with intestinal and prostate cancer who wanted a qualified second opinion. These patients are able to benefit from the expertise of centers with DKG certification [23]. These points support the importance of oncological second opinions—a topic that is attracting greater social attention and becoming a focus in health-care policy.

Obstacles that prevent patients from obtaining a second opinion still persist. After receiving a diagnosis of cancer, patients find themselves under massive time pressure to start treatment—either due to their own feelings and anxieties, or due to pressure from the attending physicians and not rarely from relatives. In addition to the excess of available information that is frequently observed nowadays, particularly from the Internet, as well as an increasing entitlement mentality, a fear of annoying the doctors providing the initial treatment can be identified as a potential obstacle to obtaining an oncological second opinion [24]. In the present study, 14.0% of the participating patients—i.e., one in seven—were concerned that the initial physicians might be annoyed about them obtaining a second opinion. Another obstacle could be that many patients are initially in a state of shock. They have a feeling that everything is collapsing under them, and they are only able to take in very limited amounts of information [24]. This may sometimes be a reason why patients are better informed after receiving an oncological second opinion than they were after the first medical consultation—because they are better able to prepare for the second opinion and can concentrate fully on information about the further procedure during the discussion, since they have already known the diagnosis for some time and have processed it to some extent.

Various approaches are possible for lowering the barriers to obtaining an oncological second opinion. In addition to a holistic approach to treatment and patient empowerment, providing written patient information, on the one hand, and improved communication with patients on the other may be an essential basis for satisfying patients’ need for information and achieving greater patient satisfaction. Patients should have the option of obtaining an oncological second opinion openly offered to them by consulting physicians, and if necessary they can decide together whether it would be useful. In this setting, patients could be encouraged to obtain a second opinion and would not have a bad conscience about being disloyal to the physicians treating them [24].

## Conclusions

An expert oncological second opinion from a physician is of particular importance for patients who have been diagnosed with breast cancer or gynecological malignancies. It can allay fears and uncertainties and increase patient satisfaction, and this, in turn, can enhance compliance over the longer term. Even a contrary second opinion is not primarily regarded as negative. In this situation, the first physician should contact the doctor who has given the second opinion and discuss treatment options on a basis of trust. It can also be recommended that if there is incomplete information or recommendations that are not understandable, the second opinion provider should contact the initial physician before patients are unsettled by any divergences.

## Electronic supplementary material

Below is the link to the electronic supplementary material.

Follow-up questionnaire (time T1) of the second opinion project of the CCC Erlangen-EMN (Germany) in their original German language versions (PDF 183 kb)

Patient questionnaire (time T0) of the second opinion project of the CCC Erlangen-EMN (Germany) in their original German language versions (PDF 227 kb)
