# Supplementary material for: Analysis of motives and patient satisfaction in oncological second opinions provided by a certified university breast and gynecological cancer center
Source: Arch Gynecol Obstet. 2020 Apr 9;301(5):1299–306. doi: 10.1007/s00404-020-05525-2 (PMC7181428; doi:10.1007/s00404-020-05525-2)
Supplement: Supplementary file 1 — Follow-up questionnaire (time T1) of the second opinion project of the CCC Erlangen-EMN (Germany) in their original German language versions (PDF 183 kb) [file 404_2020_5525_MOESM1_ESM.pdf]

Folgefragebogen zur Auswertung des
Patientinnennutzens von Zweitmeinungen
am CCC-Erlangen-EMN

Name, Vorname	
Geburtsdatum	
Aktuelles Datum	

Arztzufriedenheit

Fühlen Sie sich nach der Zweitmeinung besser informiert als vorher?	☐ Ja<br>☐ Nein
Haben sich die Ärzte am CCC Erlangen-EMN ausreichend Zeit für Sie genommen?	☐ Ja<br>☐ Nein
Konnten Sie im Rahmen der Zweitmeinung am CCC Erlangen-EMN Ihre Fragen stellen?	☐ Ja<br>☐ Nein
Wurden diese Fragen auch beantwortet? Diese Frage bitte nur beantworten, falls Sie die vorherige Frage mit „Ja" beantwortet haben.	☐ Ja<br>☐ Nein
Haben die Ärzte des CCC Erlangen-EMN ihre Informationen verständlich vermittelt?	☐ Ja<br>☐ Nein
Haben sie sich an Ihre Vorkenntnisse angepasst?	☐ Ja<br>☐ Nein
Waren Sie mit der Kommunikation zwischen den Ärzten des CCC Erlangen-EMN und Ihnen zufrieden?	☐ Ja<br>☐ Nein, warum nicht: ____________________<br>______________________________<br>______________________________
Fühlten Sie sich im Rahmen der Zweitmeinung am CCC Erlangen-EMN verstanden?	☐ Ja<br>☐ Nein
Bestand nach der Zweitmeinung weiterer Informationsbedarf und wenn ja, bezüglich welcher Themen?<br>*(Mehrfachantworten möglich)*	☐ Nein<br>☐ Ja:<br>☐ Alternative Behandlungsmöglichkeiten<br>☐ Prognose<br>☐ Diagnose<br>☐ weiteres: ____________________<br>______________________________
Fühlten Sie sich am CCC Erlangen-EMN gut aufgehoben und in kompetenten Händen?	☐ Ja<br>☐ Nein

Haben Sie nach der Beratung in Erlangen noch weitere Ärzte aufgrund Ihrer Erkrankung für eine weitere Meinung konsultiert? Wenn Ja, wie viele?	☐ Nein<br>☐ Ja<br>Anzahl: ________
Wollen Sie aktuell noch den Rat weiterer Ärzte einholen?	☐ Ja<br>☐ Nein
War Ihr erstbehandelnder Arzt verärgert, weil Sie eine Zweitmeinung eingeholt haben?	☐ Ja<br>☐ Nein<br>☐ Ich habe ihn seitdem nicht mehr gesehen.
Hat sich die Beziehung zu Ihrem erstbehandelnden Arzt nach der Zweitmeinung verändert?	☐ Nein, ich vertraue ihm immer noch so wie vorher.<br>☐ Ja, ich vertraue ihm seitdem besser.<br>☐ Ja, ich vertraue ihm seitdem weniger.<br>☐ Ja, ich vertraue ihm nicht mehr.<br>☐ weiteres: ________________________________
Wie zufrieden sind Sie insgesamt mit der Beratung am CCC Erlangen-EMN?<br>In Schulnoten: 1(sehr gut) – 6(ungenügend)	1 | 2 | 3 | 4 | 5 | 6

Therapie

War die Therapieempfehlung des CCC Erlangen-EMN aus Ihrer Sicht identisch mit der Empfehlung des erstbehandelnden Arztes?	☐ Ja<br>☐ Nein
Hatten Sie das Gefühl, dass die Ärzte am CCC Erlangen-EMN mit Ihnen mehr Therapiemöglichkeiten besprochen haben als Ihr erster Arzt?	☐ Ja, mehr ist besprochen worden.<br>☐ Nein, das Gleiche ist besprochen worden.<br>☐ Nein, weniger ist besprochen worden.
Waren Sie an der Entscheidungsfindung der endgültigen Therapie beteiligt?	☐ Ja<br>☐ Nein
Hat sich durch die Zweitmeinung der Therapievorschlag geändert?	☐ Ja<br>☐ Nein
Haben Sie sich in der Zwischenzeit für eine Therapie entschieden?	☐ Ja<br>☐ Nein
Für welche Therapie haben Sie sich entschieden?	☐ Ich habe mich noch nicht entschieden.<br>☐ Für die Therapieempfehlung des Comprehensive Cancer Center Erlangen-EMN<br>☐ Für die Therapieempfehlung des erstinformierenden Arztes<br>☐ Für die Therapieempfehlung eines anderen Arztes *(bitte Name und Anschrift nennen):*<br>____________________<br>____________________<br>____________________
Werden Sie schon behandelt? Falls Ja, wie lange schon?	☐ Nein<br>☐ Ja<br>Behandlung seit ca.: ____________
Wo lassen Sie sich behandeln oder wo werden Sie sich behandeln lassen?	☐ Ich weiß es noch nicht.<br>☐ Beim erstinformierenden Arzt<br>☐ In der Frauenklinik des Universitätsklinikums Erlangen<br>☐ In einer anderen Klinik<br>Name: ________________<br>Adresse: ________________<br>____________________<br>____________________
Wurden Ihnen im Rahmen der Zweitmeinung am CCC Erlangen-EMN komplementärmedizinische Therapiemöglichkeiten (wie z.B. Naturheilverfahren) aufgezeigt?	☐ Nein<br>☐ Ja, diese wurden zusätzlich zur eigentlichen Therapie angeboten (sogenannte „Add-ons“).<br>☐ Ja, diese wurden als alternative Therapie angeboten.

Wie stehen Sie nach der Zweitmeinung in Erlangen zu komplementärer Medizin?	☐ Ich vertraue bei der Therapie des Krebs ausschließlich auf die Schulmedizin.<br>☐ Ich habe mich inzwischen über komplementäre Behandlungsmöglichkeiten informiert.<br>☐ Ich habe mittlerweile Naturheilverfahren zur Therapie des Krebs ausprobiert.<br>☐ Ich will Komplementärmedizin zusätzlich zur geplanten Therapie ausprobieren.<br>☐ Ich will den Krebs ausschließlich mit komplementärer Medizin therapieren.
Fühlen Sie sich durch die Zweitmeinung sicherer, die richtige Entscheidung für die Therapie getroffen zu haben?	☐ Ja<br>☐ Nein<br>☐ Ich habe mich noch nicht für eine Therapie entschieden.
Haben Sie das Gefühl, dass Sie durch die Zweitmeinung weniger Zweifel bei der Durchführung der Therapie haben?	☐ Ja<br>☐ Nein<br>☐ Ich werde noch nicht therapiert.
Haben Sie den Eindruck, dass Sie durch die Zweitmeinung die Nebenwirkungen der Therapie besser verkraften können, weil Sie wissen, dass Sie sich für die richtige Therapie entschieden haben?	☐ Ja<br>☐ Nein<br>☐ Ich werde noch nicht therapiert.

Psychische Befindlichkeit

Sind Sie aus dem Gespräch mit einem zufriedenen Gefühl herausgegangen?	☐ Ja<br>☐ Nein<br>☐ Unverändert

Fragen zum Fortschreiten der Erkrankung:<br>*Im Folgenden finden Sie eine Reihe von Aussagen, die sich alle auf Ihre Erkrankung und mögliche Zukunftssorgen von Ihnen beziehen. Bitte kreuzen Sie bei jeder Aussage an, was für Sie zutrifft. Sie können wählen zwischen „nie", „selten", „manchmal", „oft" und „sehr oft". Bitte lassen Sie keine Frage aus.*<br>*Sie werden sehen, dass einige Fragen nicht auf Sie zutreffen. Wenn Sie beispielsweise keine Familie haben, können Sie Fragen zur Familie nicht beantworten. Wir bitten Sie, in diesen Fällen ein Kreuz bei „nie" zu machen.*

„Wenn ich an den weiteren Verlauf meiner Erkrankung denke, bekomme ich Angst."	☐ nie ☐ selten ☐ manchmal ☐ oft ☐ sehr oft
„Vor Arztterminen oder Kontrolluntersuchungen bin ich ganz nervös."	☐ nie ☐ selten ☐ manchmal ☐ oft ☐ sehr oft
„Ich habe Angst vor Schmerzen."	☐ nie ☐ selten ☐ manchmal ☐ oft ☐ sehr oft
„Der Gedanke, ich könnte im Beruf nicht mehr so leistungsfähig sein, macht mir Angst."	☐ nie ☐ selten ☐ manchmal ☐ oft ☐ sehr oft
„Wenn ich Angst habe, spüre ich das auch körperlich (z.B. Herzklopfen, Magenschmerzen, Verspannung)."	☐ nie ☐ selten ☐ manchmal ☐ oft ☐ sehr oft
„Die Frage, ob meine Kinder meine Krankheit auch bekommen könnten, beunruhigt mich."	☐ nie ☐ selten ☐ manchmal ☐ oft ☐ sehr oft
„Es beunruhigt mich, dass ich im Alltag auf fremde Hilfe angewiesen sein könnte."	☐ nie ☐ selten ☐ manchmal ☐ oft ☐ sehr oft
„Ich habe Sorge, dass ich meinen Hobbys wegen meiner Erkrankung irgendwann nicht mehr nachgehen kann."	☐ nie ☐ selten ☐ manchmal ☐ oft ☐ sehr oft
„Ich habe Angst vor drastischen medizinischen Maßnahmen im Verlauf der Erkrankung."	☐ nie ☐ selten ☐ manchmal ☐ oft ☐ sehr oft
„Ich mache mir Sorgen, dass meine Medikamente meinem Körper schaden könnten."	☐ nie ☐ selten ☐ manchmal ☐ oft ☐ sehr oft
„Mich beunruhigt, was aus meiner Familie wird, wenn mir etwas passieren sollte."	☐ nie ☐ selten ☐ manchmal ☐ oft ☐ sehr oft
„Der Gedanke, ich könnte wegen Krankheit in der Arbeit ausfallen, beunruhigt mich."	☐ nie ☐ selten ☐ manchmal ☐ oft ☐ sehr oft

Bitte kreisen Sie am Thermometer unten die Zahl ein, die am besten beschreibt, wie belastet Sie sich in der letzten Woche, einschließlich heute, gefühlt haben
(10 = maximal mögliche Belastung; 0 = keine Belastung).

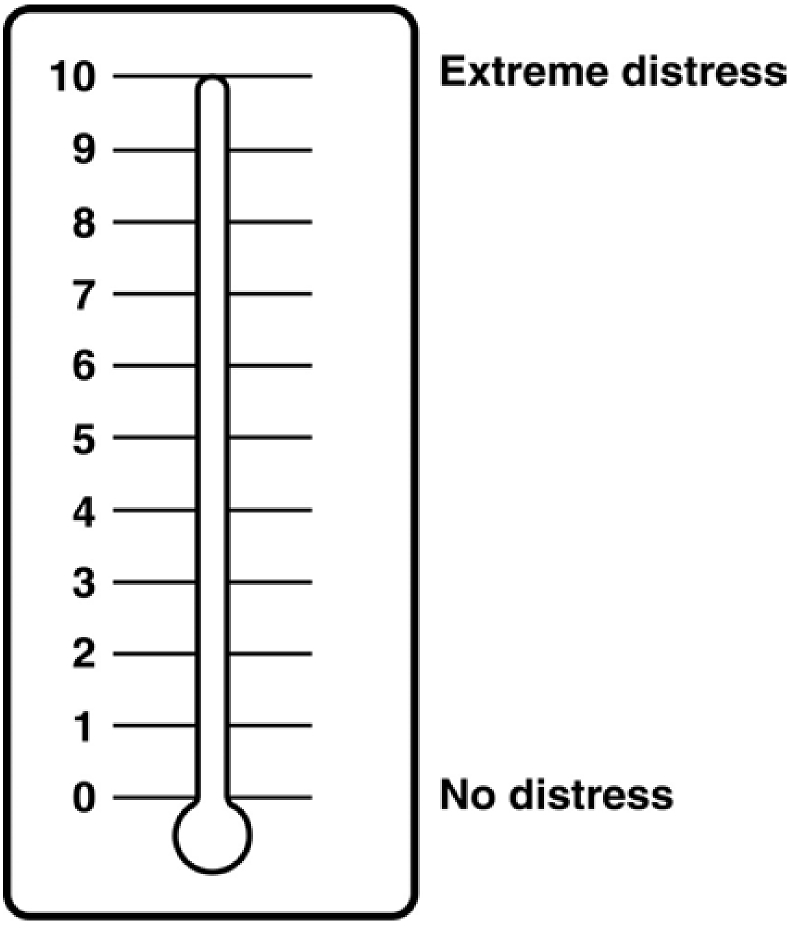

Fazit

Fanden Sie das Einholen einer Zweitmeinung generell sinnvoll?	☐ Ja<br>☐ Nein
Würden Sie anderen Frauen auch die Einholung einer Zweitmeinung im Falle einer Krebserkrankung empfehlen?	☐ Ja<br>☐ Nein
Würden Sie anderen Frauen eine Zweitmeinung am Comprehensive Cancer Center Erlangen-EMN empfehlen?	☐ Ja<br>☐ Nein

Herzlichen Dank für Ihre Teilnahme!
