# Supplementary material for: Analysis of motives and patient satisfaction in oncological second opinions provided by a certified university breast and gynecological cancer center
Source: Arch Gynecol Obstet. 2020 Apr 9;301(5):1299–306. doi: 10.1007/s00404-020-05525-2 (PMC7181428; doi:10.1007/s00404-020-05525-2)
Supplement: Supplementary file 2 — Patient questionnaire (time T0) of the second opinion project of the CCC Erlangen-EMN (Germany) in their original German language versions (PDF 227 kb) [file 404_2020_5525_MOESM2_ESM.pdf]

Einwilligungserklärung zur wissenschaftlichen Analyse Ihrer Anfrage nach einer onkologischen Zweitmeinung

Sehr geehrte Frau___________________________________,

hiermit bitten wir Sie um Ihr Einverständnis zur wissenschaftlichen Verwendung Ihrer personenbezogenen Daten aus der onkologischen Zweitmeinung, wie es Ihnen in der Patienteninformation näher erläutert worden ist.

Second Opinion Outcome am Comprehensive Cancer Center Erlangen-EMN (CCC ER-EMN)

Erfassung und Auswertung des Patientinnennutzens durch Zweitmeinung am CCC ER-EMN

Comprehensive Cancer Center Erlangen - Europäische Metropolregion Nürnberg (CCC ER-EMN)

Universitätsklinikum Erlangen

Östliche Stadtmauerstraße 30

Ansprechpartner: 09131 85 47029

A Allgemeines

Ich bin durch den Studienarzt bzw. den Krebsinformationsdienst des CCC ER-EMN über den Zweck, den Ablauf, die Bedeutung der Versorgungsforschungsstudie sowie die Vorteile und Risiken, die damit verbunden sein können, schriftlich aufgeklärt worden. Die schriftliche Patienteninformation habe ich gelesen. Alle meine Fragen sind zu meiner Zufriedenheit beantwortet worden.

Ich habe eine Kopie der Patienteninformation und Einverständniserklärung ausgehändigt bekommen. Ich hatte genügend Zeit, um meine Entscheidung zur Studienteilnahme zu überdenken und frei zu treffen.

Meine folgenden Erklärungen reichen nur so weit, wie mir dies im Rahmen der schriftlichen Patienteninformation näher dargelegt wurde. Meine folgenden Erklärungen berechtigen und verpflichten die oben einleitend genannte Institution.

B1 Einwilligung in die Anforderung und Einsicht von vorhandenen Daten zur Anforderung der onkologischen Zweitmeinung und der Entscheidung der Tumorkonferenz zur onkologischen Zweitmeinung
(zutreffendes bitte ankreuzen)

O Ich bin mit der Anforderung und Einsicht meiner Unterlagen sowie der Tumorkonferenzentscheidung zur onkologischen Zweitmeinung einverstanden.

O Ich stimme zu, dass die statistische Auswertung meiner Unterlagen und der Tumorkonferenzentscheidung in anonymisierter Form erfolgt.

O Ich stimme zu, dass die primäre Behandlungsempfehlung und die Behandlungsempfehlung der onkologischen Zweitmeinung mit der oben genannten Fragestellung verwendet werden.

  O Ich bin damit einverstanden, dass meine Unterlagen und die Tumorkonferenzentscheidung der onkologischen Zweitmeinung und die daraus ermittelten Daten nur für die eine Studie verwendet werden

  oder

  O für Studien mit **allen** wissenschaftlichen in Betracht kommenden Fragestellungen verwendet werden.

B2 Einverständnis, Fragebögen übermittelt zu bekommen
(zutreffendes bitte ankreuzen)

O Ich bin darüber hinaus damit einverstanden, Fragebögen bezüglich meiner Erwartungshaltung und meiner Patientenzufriedenheit übermittelt zu bekommen.

O Ich bin damit einverstanden, dass meine freiwilligen Aussagen im Rahmen der oben genannten Fragebögen statistisch ausgewertet werden.

  O Ich bin damit einverstanden, dass die aus den Fragebögen resultierenden Daten nur für die eine Studie verwendet werden

  oder

  O für Studien mit **allen** wissenschaftlichen in Betracht kommenden Fragestellungen verwendet werden.

B3 Unentgeltlichkeit

Ich bin mir bewusst, dass ich für die Einwilligung in die Einsicht und Auswertung meiner zur Anforderung der onkologischen Zweitmeinung notwendigen Unterlagen und für die Einsicht und Auswertung der Tumorkonferenzentscheidung, sowie für die freiwilligen Angaben im Rahmen des Fragebogens kein Entgelt erhalte.

C Datenschutzrechtliche Einwilligungserklärung

(zutreffendes bitte ankreuzen)

O Ich bin damit einverstanden, dass die einleitend genannte Person bzw. ein Mitarbeiter der einleitend genannten Institution Einblick in meine Original-Krankenunterlagen nimmt.

Ich stimme zu, dass Daten, die meine Person betreffen (hierzu gehören insbesondere auch Krankheitsdaten aus meinen Krankenunterlagen) unter der Verantwortung der oben genannten Institution in anonymisierter Form

O für Studien mit der oben genannten Fragestellung gespeichert und verarbeitet werden

oder

O für Studien mit **allen** wissenschaftlichen in Betracht kommenden Fragestellungen gespeichert und verarbeitet werden.

Widerruf der Zustimmung zur Datenverwendung

Ich weiß, dass ich meine Zustimmung zur Verwendung meiner Daten jederzeit und ohne Angabe von Gründen gegenüber der einleitend genannten Institution bzw. Person widerrufen kann und dass dies keinen Einfluss auf meine etwaige weitere ärztliche Behandlung hat.

Ich bin mir bewusst, dass im Falle einer anonymisierten Speicherung meiner Daten deren Löschung zu einem späteren Zeitpunkt nicht möglich ist.

____________	______________________________	________________________
Ort / Datum	Name der Patientin	Unterschrift

Fragebogen zur Auswertung des
Patientinnennutzens von Zweitmeinungen
am CCC-Erlangen-EMN

CCC Comprehensive Cancer Center
Erlangen-EMN
Europäische Metropolregion Nürnberg

Allgemeine Angaben

Name, Vorname	
Geburtsdatum	
Geburtsort	
Geburtsort der Eltern	
Staatsangehörigkeit	
Aktueller Familienstand	☐ Ledig<br>☐ Verheiratet<br>☐ Geschieden<br>☐ Verwitwet
Kinder	☐ Nein<br>☐ Ja (falls Ja, bitte Anzahl der Kinder und Alter des jüngsten Kindes angeben)<br>Anzahl: ___________<br>Alter des jüngsten Kindes: ___________
Bildungsstand	☐ Kein Abschluss<br>☐ Hauptschulabschluss<br>☐ Realschulabschluss<br>☐ Abitur<br>☐ Fachhochschulabschluss<br>☐ Universitätsabschluss
Berufsausbildung *(Freitext)*	
Berufstätigkeit	☐ In Ausbildung<br>☐ Erwerbstätig<br>☐ Nicht erwerbstätig (Hausfrau)<br>☐ In Elternzeit<br>☐ Im Ruhestand<br>☐ Arbeitslos<br>☐ Sonstiges: ______________________________________
Postleitzahl des Wohnorts	
Versicherungsart	☐ Gesetzlich krankenversichert (GKV)<br>☐ Privat krankenversichert (PKV)<br>☐ Selbstzahler
Menopausaler Status	☐ Vor den Wechseljahren (prämenopausal, d.h. Sie haben Ihre Regelblutung noch)<br>☐ In den Wechseljahren (perimenopausal)<br>☐ Nach den Wechseljahren (postmenopausal, d.h. Sie hatten seit einem Jahr keine Regelblutung mehr)

Informationen zur Krebserkrankung

Um welche Krebserkrankung handelt es sich bei Ihnen? *(Mehrfachnennungen möglich)*	☐ Brustkrebs<br>☐ Gebärmutterkrebs<br>☐ Gebärmutterhalskrebs<br>☐ Eierstockkrebs<br>☐ Sonstiges: ____________________
Wie lange haben Sie schon Ihre Diagnose?	☐ < 1 Monat<br>☐ zwischen 1 Monat und 6 Monaten<br>☐ zwischen 6 Monaten und 1 Jahr<br>☐ > 1 Jahr
Haben Sie bereits einen konkreten Therapievorschlag erhalten?	☐ Nein<br>☐ Ja, von *(bitte Arzt und Anschrift nennen):*<br>____________________<br>____________________<br>____________________
Wurden Ihnen mehrere unterschiedliche Therapievorschläge gemacht?	☐ Nein<br>☐ Ja, wie viele: ____________________
Waren Sie an der Entscheidungsfindung der geplanten Therapie beteiligt?	☐ Nein<br>☐ Ja
Wurden Sie schon in Bezug auf die aktuelle Diagnose vorbehandelt?	☐ Nein<br>☐ Ja, von *(bitte Arzt und Anschrift nennen):*<br>____________________<br>____________________<br>____________________
Wie stehen Sie zu alternativmedizinischen Therapiemöglichkeiten (wie z.B. Naturheilverfahren)?	☐ Ich vertraue bei der Therapie des Krebs ausschließlich auf die Schulmedizin<br>☐ Ich habe mich über alternativmedizinische Behandlungsmöglichkeiten informiert<br>☐ Ich habe bereits Alternativmedizin zur Therapie des Krebs ausprobiert<br>☐ Ich will Alternativmedizin zusätzlich zur geplanten Therapie ausprobieren<br>☐ Ich will den Krebs ausschließlich mit Alternativmedizin therapieren<br>Warum: ____________________
Hat Ihnen Ihr erstbehandelnder Arzt alternativmedizinische Therapiemöglichkeiten aufgezeigt?	☐ Nein<br>☐ Ja, diese wurden zusätzlich zur eigentlichen Therapie angeboten (sogenannte „Add-ons“)<br>☐ Ja, diese wurden als alternative Therapie angeboten

Zur Zweitmeinung

Sie haben sich für eine Zweitmeinung entschieden...	*Bitte alle Fragen beantworten*
… aufgrund Ihres schlechten Gesundheitszustandes.	☐ Ja<br>☐ Nein
… aufgrund der extremen, belastenden Situation.	☐ Ja<br>☐ Nein
… wegen der Hoffnung auf eine Änderung der Diagnose.	☐ Ja<br>☐ Nein
… wegen der Hoffnung auf eine Änderung der vorgeschlagenen Therapie.	☐ Ja<br>☐ Nein
… wegen des Wunsches nach Bestätigung der Diagnose durch eine weitere Institution.	☐ Ja<br>☐ Nein
… wegen des Wunsches nach Bestätigung der Therapie.	☐ Ja<br>☐ Nein
… aus Angst, die falsche Entscheidung bezüglich der Therapie zu treffen.	☐ Ja<br>☐ Nein
… aus Bedenken, dass die Nebenwirkungen der geplanten Maßnahmen Ihre Gesundheit zu stark beeinträchtigen (z.B. Haarausfall, Übelkeit, Schmerzen...).	☐ Ja<br>☐ Nein
... aus Angst, dass die empfohlene Therapie/empfohlenen Medikamente keine Wirkung zeigen.	☐ Ja<br>☐ Nein
…, um nichts unversucht zu lassen, die Krankheit zu besiegen.	☐ Ja<br>☐ Nein
…, weil die Meinung mehrerer Ärzte zur bestmöglichen Therapie führt.	☐ Ja<br>☐ Nein
…, weil die Ärzte hier mehr Erfahrung haben mit Krebserkrankungen.	☐ Ja<br>☐ Nein
… aus Unzufriedenheit mit dem erstinformierenden Arzt.	☐ Ja<br>☐ Nein
Sonstiges *(Freitext):*	____________________<br>____________________<br>____________________
Haben Sie sich im Internet über Ihre Krebserkrankung und mögliche Therapieoptionen erkundigt?	☐ Ja<br>☐ Nein
Wie haben Sie von der Möglichkeit der Zweitmeinung erfahren?<br>*(Mehrfachantworten möglich)*	☐ Behandelnder Arzt<br>☐ Internet<br>☐ Krankenkasse<br>☐ Angehörige/Verwandte<br>☐ Freunde/Bekannte<br>☐ Sonstiges: ____________________

Von wem ging der Anreiz zur Zweitmeinung aus?<br>*(Mehrfachantworten möglich)*	☐ Von Ihnen selbst<br>☐ Partner<br>☐ Kinder<br>☐ Freunde/Bekannte<br>☐ Hausarzt<br>☐ Niedergelassener Gynäkologe<br>☐ Von dem Arzt, der die Erstempfehlung durchgeführt hat<br>☐ Sonstiges: ___________________________
Haben Sie bezüglich Ihrer aktuellen Diagnose bereits mehrere Zweitmeinungen eingeholt? Falls Ja, bitte die Anzahl angeben.	☐ Nein<br>☐ Ja<br>Anzahl der Zweitmeinungen: ____________
Warum haben Sie sich für eine Zweitmeinung am Comprehensive Cancer Center Erlangen-EMN entschieden?<br>*(Mehrfachantworten möglich)*	☐ weil das Klinikum Erlangen eine Universitätsklinik ist<br>☐ weil das Universitätsklinikum auch ein Onkologisches Spitzenzentrum ist (Comprehensive Cancer Center, CCC)<br>☐ Guter Ruf der behandelnden Ärzte<br>☐ Nähe zum Wohnort<br>☐ Empfehlung von Freunden/Bekannten<br>☐ Empfehlung Ihrer Versicherung<br>☐ Auf Anraten des Hausarztes<br>☐ Auf Anraten eines niedergelassenen Gynäkologen<br>☐ Auf Anraten des Arztes, der die Erstempfehlung durchgeführt hat<br>☐ Sonstiges: ___________________________
Haben Sie grundsätzlich ein hohes Informationsbedürfnis?	☐ Ja<br>☐ Nein
Sind Sie in Ihrer Grundhaltung eher skeptisch eingestellt?	☐ Ja<br>☐ Nein

Arztzufriedenheit

Weiß Ihr erstbehandelnder Arzt, dass Sie eine Zweitmeinung einholen?	☐ Ja<br>☐ Nein
Haben Sie Sorge davor, dass die Einholung einer Zweitmeinung die Beziehung zu Ihrem erstbehandelnden Arzt verändern könnte?	☐ Ja<br>☐ Nein
Haben Sie Sorge, dass Ihr erstbehandelnder Arzt aufgrund der Zweitmeinung verärgert sein wird?	☐ Ja<br>☐ Nein
Hat sich Ihr erstbehandelnder Arzt für Sie Zeit genommen hat, oder war er eher unter Zeitdruck?	☐ Ja<br>☐ Nein, unter Zeitdruck
War Ihr erstbehandelnder Arzt einfühlsam beim Überbringen der Diagnose?	☐ Ja<br>☐ Nein
Hatten Sie Zeit, ihm Fragen zu stellen?	☐ Ja<br>☐ Nein
Hat er Ihnen diese Fragen auch beantwortet? Diese Frage bitte nur beantworten, falls Sie die vorherige Frage mit „Ja“ beantwortet haben.	☐ Ja<br>☐ Nein
Hat Ihr erstbehandelnder Arzt seine Informationen verständlich vermittelt?	☐ Ja<br>☐ Nein
Hat er sich an Ihre Vorkenntnisse angepasst?	☐ Ja<br>☐ Nein
Wurde Ihnen Infomaterial zur Verfügung gestellt?	☐ Ja<br>☐ Nein
Wurden Ihnen Behandlungsalternativen aufgezeigt?	☐ Ja<br>☐ Nein
Wurden unterschiedliche Behandlungsmöglichkeiten von Ihrem erstbehandelnden Arzt mit Ihnen diskutiert? Falls Ja, welche?	☐ Nein<br>☐ Ja<br>Besprochene Behandlungsmöglichkeiten:<br>____________________<br>____________________<br>____________________
Waren Sie mit der Kommunikation zwischen Ihrem erstbehandelnden Arzt und Ihnen zufrieden?	☐ Ja<br>☐ Nein, warum nicht: ____________________<br>____________________
Besteht bei Ihnen nach der Beratung durch Ihren erstbehandelnden Arzt weiterer Informationsbedarf und wenn ja, bezüglich welcher Themen?<br>*(Mehrfachantworten möglich)*	☐ Nein<br>☐ Ja:<br>☐ Alternative Behandlungsmöglichkeiten<br>☐ Prognose<br>☐ Diagnose<br>☐ Sonstiges: ____________________<br>____________________
Sind Sie insgesamt mir Ihrem erstbehandelnden Arzt zufrieden?	☐ Ja<br>☐ Nein
Vertrauen Sie Ihrem Arzt in jeglicher Hinsicht?	☐ Ja<br>☐ Nein

Psychische Befindlichkeit

Hat die Diagnose „Krebs“ Ihr Leben verändert?	☐ Nein, ich lebe weiter so wie bisher.<br>☐ Ja, ich lebe bewusster und genieße mein Leben mehr.<br>☐ Ja, ich bin sehr belastet und leide sehr unter meiner Erkrankung.<br>☐ Sonstiges: ___________________________

Fragen zum Fortschreiten der Erkrankung:
Im Folgenden finden Sie eine Reihe von Aussagen, die sich alle auf Ihre Erkrankung und mögliche Zukunftssorgen von Ihnen beziehen. Bitte kreuzen Sie bei jeder Aussage an, was für Sie zutrifft. Sie können wählen zwischen „nie“, „selten“, „manchmal“, „oft“ und „sehr oft“. Bitte lassen Sie keine Frage aus.
Sie werden sehen, dass einige Fragen nicht auf Sie zutreffen. Wenn Sie beispielsweise keine Familie haben, können Sie Fragen zur Familie nicht beantworten. Wir bitten Sie, in diesen Fällen ein Kreuz bei „nie“ zu machen.

„Wenn ich an den weiteren Verlauf meiner Erkrankung denke, bekomme ich Angst.“	☐ nie ☐ selten ☐ manchmal ☐ oft ☐ sehr oft
„Vor Arztterminen oder Kontrolluntersuchungen bin ich ganz nervös.“	☐ nie ☐ selten ☐ manchmal ☐ oft ☐ sehr oft
„Ich habe Angst vor Schmerzen.“	☐ nie ☐ selten ☐ manchmal ☐ oft ☐ sehr oft
„Der Gedanke, ich könnte im Beruf nicht mehr so leistungsfähig sein, macht mir Angst.“	☐ nie ☐ selten ☐ manchmal ☐ oft ☐ sehr oft
„Wenn ich Angst habe, spüre ich das auch körperlich (z.B. Herzklopfen, Magenschmerzen, Verspannung).“	☐ nie ☐ selten ☐ manchmal ☐ oft ☐ sehr oft
„Die Frage, ob meine Kinder meine Krankheit auch bekommen könnten, beunruhigt mich.“	☐ nie ☐ selten ☐ manchmal ☐ oft ☐ sehr oft
„Es beunruhigt mich, dass ich im Alltag auf fremde Hilfe angewiesen sein könnte.“	☐ nie ☐ selten ☐ manchmal ☐ oft ☐ sehr oft
„Ich habe Sorge, dass ich meinen Hobbys wegen meiner Erkrankung irgendwann nicht mehr nachgehen kann.“	☐ nie ☐ selten ☐ manchmal ☐ oft ☐ sehr oft
„Ich habe Angst vor drastischen medizinischen Maßnahmen im Verlauf der Erkrankung.“	☐ nie ☐ selten ☐ manchmal ☐ oft ☐ sehr oft
„Ich mache mir Sorgen, dass meine Medikamente meinem Körper schaden könnten.“	☐ nie ☐ selten ☐ manchmal ☐ oft ☐ sehr oft
„Mich beunruhigt, was aus meiner Familie wird, wenn mir etwas passieren sollte.“	☐ nie ☐ selten ☐ manchmal ☐ oft ☐ sehr oft
„Der Gedanke, ich könnte wegen Krankheit in der Arbeit ausfallen, beunruhigt mich.“	☐ nie ☐ selten ☐ manchmal ☐ oft ☐ sehr oft

Fragen zur Erfassung Ihrer psychosozialen Belastung:
Bitte geben Sie an, ob Sie in einem der nachfolgenden Bereiche in der letzten Woche, einschließlich heute, Probleme hatten. Kreuzen Sie für jeden Bereich JA oder NEIN an.

Praktische Probleme bezüglich:	
Wohnsituation	☐ Ja ☐ Nein
Versicherung	☐ Ja ☐ Nein
Arbeit/Schule	☐ Ja ☐ Nein
Beförderung (Transport)	☐ Ja ☐ Nein
Kinderbetreuung	☐ Ja ☐ Nein
Familiäre Probleme	
Im Umgang mit dem Partner	☐ Ja ☐ Nein
Im Umgang mit den Kindern	☐ Ja ☐ Nein
Emotionale Probleme	
Sorgen	☐ Ja ☐ Nein
Ängste	☐ Ja ☐ Nein
Traurigkeit	☐ Ja ☐ Nein
Depression	☐ Ja ☐ Nein
Nervosität	☐ Ja ☐ Nein
Verlust des Interesses an alltäglichen Aktivitäten	☐ Ja ☐ Nein
Spirituelle/religiöse Belange	
In Bezug auf Gott	☐ Ja ☐ Nein
Verlust des Glaubens	☐ Ja ☐ Nein
Körperliche Probleme	
Schmerzen	☐ Ja ☐ Nein
Übelkeit	☐ Ja ☐ Nein
Erschöpfung	☐ Ja ☐ Nein
Schlaf	☐ Ja ☐ Nein
Bewegung/Mobilität	☐ Ja ☐ Nein
Waschen, Ankleiden	☐ Ja ☐ Nein
Äußeres Erscheinungsbild	☐ Ja ☐ Nein
Atmung	☐ Ja ☐ Nein
Entzündungen im Mundbereich	☐ Ja ☐ Nein
Essen/Ernährung	☐ Ja ☐ Nein
Verdauungsstörungen	☐ Ja ☐ Nein
Verstopfung	☐ Ja ☐ Nein
Durchfall	☐ Ja ☐ Nein
Veränderungen beim Wasser lassen	☐ Ja ☐ Nein
Fieber	☐ Ja ☐ Nein

Trockene/juckende Haut	☐ Ja ☐ Nein
Trockene/verstopfte Nase	☐ Ja ☐ Nein
Kribbeln in Händen/Füßen	☐ Ja ☐ Nein
Angeschwollen/aufgedunsen fühlen	☐ Ja ☐ Nein
Gedächtnis/Konzentration	☐ Ja ☐ Nein
Sexuelle Probleme	☐ Ja ☐ Nein

Bitte kreisen Sie am Thermometer unten die Zahl ein, die am besten beschreibt, wie belastet Sie sich in der letzten Woche, einschließlich heute, gefühlt haben
(10 = maximal mögliche Belastung; 0 = keine Belastung).

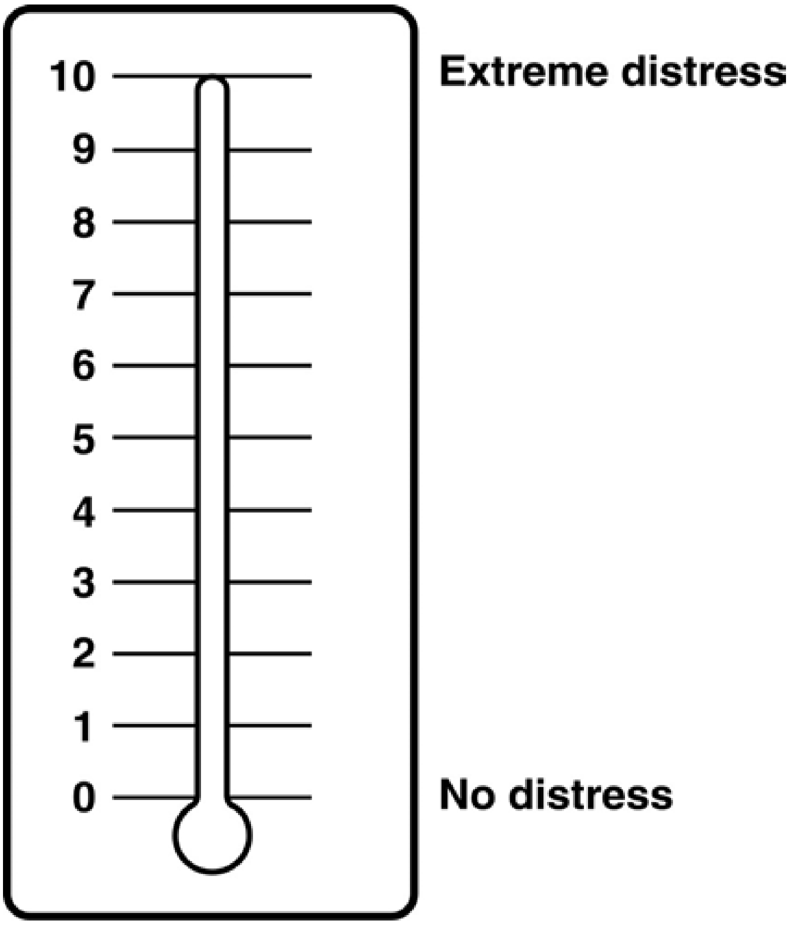

Fragen zu Ihrer seelischen Widerstandsfähigkeit:
Die folgenden Fragen beziehen sich auf verschiedene Aspekte Ihres Lebens. Jeder Satz stellt eine Frage dar, die man individuell verschieden beantworten kann. Bitte kreuzen Sie bei jeder der Aussagen an, welche Abstufung zwischen 1 und 7 Ihrer persönlichen Einstellung am ehesten entspricht. Geben Sie bitte auf jede Frage nur eine Antwort.

Haben Sie das Gefühl, dass es Ihnen ziemlich gleichgültig ist, was um Sie herum passiert?	selten/ nie	1	2	3	4	5	6	7	sehr oft
Ist es in der Vergangenheit vorgekommen, dass Sie vom Verhalten von Menschen überrascht waren, die Sie gut zu kennen glaubten?	Das ist nie passiert.	1	2	3	4	5	6	7	Das ist immer wieder passiert.
Ist es vorgekommen, dass Sie von Menschen enttäuscht wurden, auf die Sie gezählt hatten?	Das ist nie passiert.	1	2	3	4	5	6	7	Das ist immer wieder passiert.
Bis jetzt hatte Ihr Leben...	...überhaupt keine klaren Ziele.	1	2	3	4	5	6	7	...sehr klare Ziele.
Haben Sie das Gefühl, dass Sie ungerecht behandelt werden?	sehr oft	1	2	3	4	5	6	7	sehr selten/nie
Haben Sie das Gefühl, dass Sie in einer ungewohnten Situation sind und nicht wissen, was Sie tun sollen?	sehr oft	1	2	3	4	5	6	7	sehr selten/nie
Die Dinge, die Sie täglich tun, sind für Sie eine Quelle...	...tiefer Freude und Befriedigung.	1	2	3	4	5	6	7	...von Schmerz und Langeweile.
Wie oft sind Ihre Gefühle und Gedanken ganz durcheinander?	sehr oft	1	2	3	4	5	6	7	sehr selten/nie
Kommt es vor, dass Sie Gefühle in sich haben, die Sie lieber nicht spüren würden?	sehr oft	1	2	3	4	5	6	7	sehr selten/nie
Viele Leute – auch solche mit starkem Charakter – fühlen sich in bestimmten Situationen als traurige Verlierer. Wie oft haben Sie sich in der Vergangenheit so gefühlt?	nie	1	2	3	4	5	6	7	sehr oft
Wenn etwas passierte, hatten Sie im Allgemeinen den Eindruck, dass Sie dessen Bedeutung...	...über- oder unter-schätzten.	1	2	3	4	5	6	7	...richtig ein-schätzten.
Wie oft haben Sie das Gefühl, dass die Dinge, die Sie im täglichen Leben tun, eigentlich wenig Sinn haben?	sehr oft	1	2	3	4	5	6	7	sehr selten/nie
Wie oft haben Sie Gefühle, bei denen Sie sich nicht sicher sind, ob Sie die unter Kontrolle halten können?	sehr oft	1	2	3	4	5	6	7	sehr selten/nie

Herzlichen Dank für Ihre Teilnahme!
